# Expanding the clinical-pathological and genetic spectrum of *RYR1*-related congenital myopathies with cores and minicores: an Italian population study

**DOI:** 10.1186/s40478-022-01357-0

**Published:** 2022-04-15

**Authors:** Aurora Fusto, Denise Cassandrini, Chiara Fiorillo, Valentina Codemo, Guja Astrea, Adele D’Amico, Lorenzo Maggi, Francesca Magri, Marika Pane, Giorgio Tasca, Daniele Sabbatini, Luca Bello, Roberta Battini, Pia Bernasconi, Fabiana Fattori, Enrico Silvio Bertini, Giacomo Comi, Sonia Messina, Tiziana Mongini, Isabella Moroni, Chiara Panicucci, Angela Berardinelli, Alice Donati, Vincenzo Nigro, Antonella Pini, Melania Giannotta, Claudia Dosi, Enzo Ricci, Eugenio Mercuri, Giovanni Minervini, Silvio Tosatto, Filippo Santorelli, Claudio Bruno, Elena Pegoraro

**Affiliations:** 1 Department of Neurosciences DNS, University of Padova, 35128 Padua, Italy; 2 Molecular Medicine Unit, IRCCS Fondazione Stella Maris, 56128 Pisa, Italy; 3 Paediatric Neurology and Neuromuscular Disorders Unit, IRCCS Istituto Giannina Gaslini, Department of Neurosciences, Rehabilitation, Ophthalmology, Genetics, Maternal and Child Health, University of Genova, 16147 Genoa, Italy; 4 Department of Neuroscience, IRCCS Fondazione Stella Maris, 56128 Pisa, Italy; 5 Molecular Medicine Unit, Ospedale Bambin Gesù, 00165 Rome, Italy; 6 Neuroimmunology and Neuromuscular Disorders Unit, Foundation IRCCS Neurological Institute “C. Besta”, 20133 Milan, Italy; 7 Dino Ferrari Centre, Department of Neurological Sciences, University of Milan, I.R.C.C.S. Foundation Cà Granda, Ospedale Maggiore Policlinico, 20122 Milan, Italy; 8 Department of Paediatric Neurology, Catholic University, 00165 Rome, Italy; 9 Unità Operativa Complessa Di Neurologia, Fondazione Policlinico Universitario A. Gemelli IRCCS, 00168 Rome, Italy; 10 Department of Neurosciences, Psychiatry and Anaesthesiology, University of Messina, 98122 Messina, Italy; 11 SG. Battista Hospital, Neuromuscular Center, University of Turin, 10124 Turin, Italy; 12 Child Neurology Department, Neurological Institute C. Besta Foundation IRCCS, 20133 Milan, Italy; 13 Center of Translational and Experimental Myology, IRCCS Istituto Giannina Gaslini, Department of Neurosciences, Rehabilitation, Ophthalmology, Genetics, Maternal and Child Health, University of Genova, 16147 Genova, Italy; 14 Child and Adolescent Unit, IRCCS C. Mondino Foundation, 27100 Pavia, Italy; 15 Metabolic Disease Unit, AOU Meyer Children Hospital, 50139 Florence, Italy; 16 “Luigi Vanvitelli” University and Telethon Institute of Genetics and Medicine (TIGEM), 80078 Naples, Italy; 17 Child Neurology and Psychiatry Unit, IRCCS Istituto Delle Scienze Neurologiche Di Bologna, 40139 Bologna, Italy; 18 Department of Biomedical Sciences, University of Padova, 35128 Padua, Italy

**Keywords:** RYR1-related myopathies, Central core disease, Multi-minicore disease, Genotype–phenotype correlations, Neuromuscular disorder, Protein modelling

## Abstract

Mutations in the *RYR1* gene, encoding ryanodine receptor 1 (RyR1), are a well-known cause of Central Core Disease (CCD) and Multi-minicore Disease (MmD). We screened a cohort of 153 patients carrying an histopathological diagnosis of core myopathy (cores and minicores) for *RYR1* mutation. At least one *RYR1* mutation was identified in 69 of them and these patients were further studied. Clinical and histopathological features were collected. Clinical phenotype was highly heterogeneous ranging from asymptomatic or paucisymptomatic hyperCKemia to severe muscle weakness and skeletal deformity with loss of ambulation. Sixty-eight *RYR1* mutations, generally missense, were identified, of which 16 were novel. The combined analysis of the clinical presentation, disease progression and the structural bioinformatic analyses of *RYR1* allowed to associate some phenotypes to mutations in specific domains. In addition, this study highlighted the structural bioinformatics potential in the prediction of the pathogenicity of *RYR1* mutations. Further improvement in the comprehension of genotype–phenotype relationship of core myopathies can be expected in the next future: the actual lack of the human RyR1 crystal structure paired with the presence of large intrinsically disordered regions in RyR1, and the frequent presence of more than one *RYR1* mutation in core myopathy patients, require designing novel investigation strategies to completely address RyR1 mutation effect.

**Supplementary Information:**

The online version contains supplementary material available at 10.1186/s40478-022-01357-0.

## Introduction

Core myopathy are clinically and genetically heterogenous congenital myopathies defined by the presence on skeletal muscle biopsy of “cores” with focally reduced oxidative activity, lack of mitochondria and variable degrees of myofibrillar disruption [1]. Based on the histological findings on muscle biopsy, core myopathies were traditionally classified in Central Core Disease (CCD), where the cores, in transverse sections of the myofibers, are single in the centre of the fibre and span through all the length of type 1 muscle fibre, and Multi-minicore Disease (MmD) with multiple and less defined cores without specificity for fibre type [2, 3]. This binary classification of core myopathies is becoming reductive since in the same muscle biopsy both CC and Mm may exist, a transition of the main histopathology features [4] or the presence with cores of other structural abnormalities in muscle fibres (i.e. rods [5, 6]) is common. To add to this complexity, new entities are entering in the picture. The Dusty Core Disease (DuCD) is quite recent, and it is defined by irregular areas of myofibrillar disorganization with reddish-purple granular material depositions that show uneven oxidative staining and are devoid of ATPase activity [7]. Dusty cores, in contrast with central cores, have no clear borders and are not round/ovoidal in shape [7].

The core myopathies are genetically heterogeneous and histopathology may suggest the underlying gene defect only in CCD, mostly due to the ryanodine receptor 1 gene (*RYR1*), and DuCD due to biallelic *RYR1* mutations. In cores-rods myopathy and MmD mutations in a mixture of different genes have been reported including *RYR1* [5], *SELENON*, *MYH2*, *MYH7*, *TTN*, *CCDC78*, *UNC45B*, *ACTN2*, and *MEGF10*, *NEB*, *ACTA1*, *KBTBD13*, *CFL2*, *TRIP4*, and *TNNT1*[8].

CCD is clinically characterized by hypotonia, motor developmental delay and generalized muscle weakness of variable degree. Distribution of weakness is typically proximal, with prominent involvement of the hip girdle and axial muscles. Bone and joint disorders, such as congenital hip dislocation, kyphoscoliosis, pes cavus, thoracic deformities, joint contractures are often present. The age of onset is extremely variable, ranging from foetal akinesia—which has been primarily reported associated with recessive inheritance [9], or birth onset—with hypotonia and floppy infant syndrome, childhood onset with motor developmental delay, but also adult onset with variable clinical presentation, often within the same family [3]. The penetrance of the disease is variable and genotype–phenotype correlations have yet to be fully clarified [10]. The association with malignant hyperthermia (MH), a life-threatening pharmaco-genetic disorder triggered by exposure of susceptible individuals to inhalational anesthetics and succinylcholine, is strong, though variable, and all patients suffering from core myopathies should be considered susceptible [11].

Both CCD and MH are primarily caused by mutations in *RYR1*, which encodes ryanodine receptor 1 (RyR1). RyR1, located on the sarcoplasmic reticulum membrane, is a Ca^2+^ channel comprising of four identical subunits [12], each one of 5,038 amino acids, with a molecular weight of 563.5 kDa [13]. The monomers delimit a central ion-conducting pore. RyR1, interacts with the dihydropyridine receptor (DHPR), and it is involved in the excitation contraction coupling, that ensures skeletal muscle contraction upon stimulation [14, 15].

The RyR1 protein is made of a cytoplasmic shell (also known as “foot”) and domains constituting the RyR1 channel and the activation core. The foot comprises: the N-terminal domains (NTD-A, NTD-B, NTD-C), the bridge solenoid (BSol), the junctional solenoid (JSol)—which connects NTD-C to BSol, the SP1a/ryanodine receptor domains (SPRY1-SPRY3) and the RyR repeats pairs (RY1&2, RY3&4). The RyR1 channel portion and the activation core comprise several structural domains: the shell-core linker peptide, CaM, and JSol binding sites (SCLP), the core solenoid (CSol), the thumb and forefingers domain (TaF), the auxiliary transmembrane helices (TMx), the pseudo voltage sensor domain (pVSD), the channel pore domain, made of six transmembrane helices, and the C-terminal domain (CTD) [16]. This region is directly responsible for the Ca^2+^-mediated channel activation [12, 17]. Moreover, given its crucial role in muscle physiology, RyR1 activity is finely regulated through a wide variety of post-translational modifications of the channel, including oxidation, phosphorylation[18], and the binding of ligands. In particular, calmodulin (CaM), calstabin1 (previously known as FK506-binding protein 12, FKBP12) and CLIC2[19] interact with the CTD, and triadin, junctin, and calsequestrin with the cytoplasmic shell [12, 19].

Autosomal dominant *RYR1* mutations are identified in approximately 90% of CCD and are mostly localized in three hotspots: the cytoplasmic N-terminus (hotspot 1; amino acid 35–614), the central domain (hotspot 2; amino acid 2163–2458) and the C-terminus (hotspot 3; amino acid 4550- 4940) [3, 20]. On the basis of functional studies, distinct molecular mechanisms were proposed to explain how specific *RYR1* mutations could result in core myopathies and/or in MH [21]. The clinical spectrum of MmD is also variable and depends on the genetic background [22]. MmD patients may manifest hypotonia and proximal muscle weakness, ophthalmoparesis, atrophy of the small muscles of the hands. Severe neonatal-onset form have been identified [22–25].

MmD may be associated with recessive mutations in the selenoprotein N1 gene (*SEPN1*, also known as *SELENON*) [26],—as well as other genes such as *MYH2* [27, 28]*, UNC45B* [29]*, MYH7* [30], *TTN* [31], *MEGF10* [32], *SECISBP2*, *ACTA1*, *ACTN2*, *CCD78* [33], and *FXR1* [34]—but mutations in *RYR1* had been increasingly described [22, 23, 25, 35]. Here we report a large series of Italian patients affected by *RYR1-*related core myopathy characterized at the clinical, histopathological and molecular level. Structural in silico modelling of RyR1 to predict pathogenicity of the identified *RYR1* mutations has been utilized for in-depth genotype–phenotype correlations.

## Materials and methods

### Patients

An Italian network of tertiary referral Centers for congenital myopathies had been established in 2008 to collect detailed clinical, morphological, and genetic data in a large group of Italian patients. One hundred fifty-three patients were selected according to muscle histopathology consistent with cores or minicores myopathy and chosen for *RYR1* mutations analyses. Ethics committees of all participating centers approved the study and written informed consent was obtained from all patients or their legal guardians, in accordance with the ethical standards of the 1975 Declaration of Helsinki, revised in 2013. Clinical evaluation was performed at each referring Center according to a standardized protocol, including the collection of family history, disease onset and progression data, functional abilities, muscle strength and severity of contractures when present. Electrocardiogram, echocardiogram, pulmonary function test results were collected for each patient. Based on disease severity, patients were classified as: “asymptomatic” if they came to medical attention for CK elevation, without symptoms of neuromuscular disease; “paucisymptomatic” if they complained of myalgias, and/or muscle cramps but not overt muscle weakness. In this subset of patients, the presence of minor osteoarticular alterations was allowed; “myopathic” if muscle weakness was present. This last subset was further categorized according to the MRC score of proximal and distal muscles in the upper and lower limbs in mild (MRC score ≥ 4 in all muscle tested), moderate (MRC ≤ 3 in one proximal muscle) and severe (MRC ≤ 3 in two or more proximal muscles). The age of onset in symptomatic patients was defined as “congenital” if clinical symptoms appeared before 12 months of age; “early” if symptoms were present before age of 20; “adult” if symptoms appeared after 20 years of age.

### Muscle biopsy analysis

Muscle biopsies, done at the time of diagnosis, were reviewed, when available, at each referring centre by an expert myopathologist. Transverse cryosections of muscle biopsies stained with NADH-TR (nicotinamide adenine dinucleotide dehydrogenase-tetrazolium reductase), COX (cytochrome C oxidase) or SDH (succinate dehydrogenase), haematoxylin–eosin or Gomori’s trichrome were examined on upright microscope (Olympus BX60, Tokyo, Japan). All fibres with central nuclei, cores in type 1 or type 2 muscle fibres, rods, and the number of type 1 fibres were counted in 3 independent microscopic fields (at a magnification of 10$\times$). The results had been expressed as mean percentage.

### *RYR1* mutations analysis

Genomic DNA was extracted from peripheral blood according to standard procedure. The entire coding sequence of *RYR1* (NG_008866.1) was amplified from patient genomic DNA and analysed by Sanger sequencing. Frequency of mutation in the hotspots was assessed with proportion test performed with R v. 3.5.3.

### Homology modelling of the human RyR1 3D structure

The human RyR1 sequence (accession code: P21817) was obtained from Uniprot [36] and used to perform a Blastp search against the Protein Data Bank [37]. The 3D structure of the RyR1 orthologous protein from Oryctolagus cuniculus (PDB ID 5T15; electron microscopy structure, resolved at 3.6 Å) was selected as a template to build the model based on its high sequence identity and coverage with the human RyR1 (identities = 96.91%, coverage = 72%). As this structure presents multiple not resolved segments, the human RyR1 sequence was aligned against the template with Jalview [38] using T-Coffee [39] algorithm (default parameters) to identify the protein domains. Six comparative models of the target sequence were built by MODELLER [40] using the model single module. Models were then evaluated for value of discrete optimized protein energy using the DOPE method integrated in MODELLER. Model number 2 was selected as the best final model. Overall structure quality was further assessed with QMEANDisCo [41] and MolProbity [42]. The MobiDB-lite [43] was used to exactly identify and map intrinsically disordered regions boundaries mostly overlapping segments not resolved in the template structure. The model presents a good geometry with 90.09% of residues in the most Ramachandran favoured regions while the 2.05% are marked as outliers. The Rama distribution Z-score calculated for the entire model is -0.98 ± 0.11 (Goal: < 2) whereas the number of bad bonds is estimated to 0.01% of total. To avoid introduction of unwanted artifacts there was no attempt made to manually adjust the backbone torsion angles. The final 3D model corresponds to the human RyR1 segments spanning residues 24–1272, 1432–1924, 2057–2562, 2734–2940, 3645–4250, 4546–5034.

### Bioinformatics analyses of *RYR1* mutations

An integrative in silico pipeline was used to evaluate pathogenic impact of *RYR1* mutations described in Additional file 1: Fig. S1. In details, a total of 45 RyR1 homologous sequences were retrieved by PSI-BLAST [44] search (default parameters) against the UniProt sequence database and aligned with T-Coffe (default parameters) [39] to derive a conservation score used each residue. This conservation score was then used as a preliminary filter to evaluate impact of substitutions found in patients. The ELM [45] database was used to search and map functional linear motifs, while FELLS [46] was used to derive structure features. Structural inspection was performed with Chimera [47], while effect of mutations on the human RyR1 3D structure was evaluated with BLUUES [48] and RING2.0 [49]. Stability and pathogenicity assessment were carried out using a consensus approach including predictions from Polyphen2.0 [50], SIFT [51], Mupro [52], Mutationassessor [53] (Additional file 1: Fig. S1). The new Intronic variant has been analysed with two independent algorithms for splice signal detection: NetGene2 (http://www.cbs.dtu.dk/services/NetGene2/) and Berkeley Drosophila Genome Project (BDGP, https://www.fruitfly.org/seq_tools/splice.html).

### Statistical analyses

In order to evaluate associations between the presence of mutations in specific domains and several phenotypic traits (foetal hypokinesia, hypotonia at birth, arthrogryposis, respiratory insufficiency, dysphagia, delayed independent ambulation, congenital hip dislocation, facial weakness, muscle wasting, ocular involvement, bulbar involvement, contractures, foot deformities, scoliosis/rigid spine, cardiac abnormalities, cognitive issues), for each pair of protein domain and phenotypical trait, a 2 × 2 table was devised, with rows indicating presence vs. absence of mutations in that domain, and columns indicating presence vs. absence of the phenotypic trait. P-values < 0.05 are reported (without correction for multiple testing) and should be interpreted as descriptive.

## Results

At least one *RYR1* mutation was identified in 69 core myopathy patients and these have been further studied (Table 1).

**Table 1:** Genetic details of *RYR1*-affected individuals

ID/Family	Nucleotide change	Amino acid change	Family history	Exon/Intro	Affected domain	Variant classification^a^	References
1/1	c.212C > A	p.Ser71Tyr	No	E 3	NTD-A	Pathogenic	[54, 55]
	c.6847A > C	p.Asn2283His		E 42	BSol	Pathogenic	[54]
2/2	c.467G > A	p.Arg156Lys	No	E 6	NTD-A	Pathogenic	[55, 56]
3/3	c.487C > T	p.Arg163Cys	AD	E 6	NTD-A	Pathogenic	[57, 58]
4/3	c.487C > T	p.Arg163Cys	AD	E 6	NTD-A	Pathogenic	[57, 58]
5/3	c.487C > T	p.Arg163Cys	AD	E 6	NTD-A	Pathogenic	[57, 58]
6/4	c.487C > T	p.Arg163Cys	AD	E 6	NTD-A	Pathogenic	[57, 58]
7/5	c.1021G > A	p.Gly341Arg	AR	E 11	NTD-B	Pathogenic	[59, 60]
	c.1021G > A	p.Gly341Arg		E 11	NTD-B	Pathogenic	[59, 60]
8/6	c.1209C > G	p.Ile403Met	No	E 12	NTD-B	Pathogenic	[61]
9/7	c.1250 T > C	p.Leu417Pro	No	E 13	NTD-C	Likely pathogenic	[62]
10/8	c.1840C > T	p.Arg614Cys	AD	E 17	NTD-C	Pathogenic	[63, 64]
11/9	c.3301G > A	p.Val1101Met	No	E 25	SPRY2/SPRY3	Likely pathogenic	[65]
	c.14473C > T	p.Arg4825Cys		E 100	Pore	Pathogenic	[66]
12/10	c.4711A > G	p.Ile1571Val	No	E 33	SPRY2/SPRY3	Likely benign	[62, 67]
	c.7373G > A	p.Arg2458His		E 46	BSol	Pathogenic	[68]
	c.10097G > A	p.Arg3366His		E 67	BSol	Likely pathogenic	[67]
	c.10259 + 7G > A	p.( =)		I 67	BSol	Benign	dbSNP: rs143752962
	c.11798A > G	p.Tyr3933Cys		E 86	CSol	Pathogenic	[67, 69]
13/11	c.4711A > G	p.Ile1571Val	No	E 33	SPRY2/SPRY3	Likely benign	[62, 67]
	c.10097G > A	p.Arg3366His		E 67	BSol	Likely pathogenic	[67]
	c.11708G > A	p.Arg3903Gln		E 85	CSol	Pathogenic	[55]
	c.11798A > G	p.Tyr3933Cys		E 85	CSol	Pathogenic	[67, 69]
14/12	c.4816C > T	p.Arg1606Cys	No	E 33	SPRY2/SPRY3	Pathogenic	[70]
15/13	c.5510A > C	p.Gln1837Pro	AD	E 34	JSol	Pathogenic	This paper
16/14	c.6488G > A	p.Arg2163His	No	E 39	BSol	Pathogenic	[57]
17/15	c.6502G > A	p.Val2168Met	AR	E 39	BSol	Pathogenic	[60]
	c.7372C > T	p.Arg2458Cys		E 46	BSol	Pathogenic	[68]
18/16	c.7025A > G	p.Asn2342Ser	No	E 43	BSol	Pathogenic	[71]
	c.14659C > T	p.His4887Tyr		E 102	Pore	Pathogenic	[72]
19/17	c.7048G > A	p.Ala2350Thr	AD	E 44	BSol	Pathogenic	[73]
20/17	c.7048G > A	p.Ala2350Thr	AD	E 44	BSol	Pathogenic	[73]
21/18	c.7085A > G	p.Glu2362Gly	AD	E 44	BSol	Pathogenic	[55]
	c.13513G > C	p.Asp4505His		E 92	pVSD	Pathogenic	[74, 75]
22/19	c.7304G > A	p.Arg2435His	AD	E 45	BSol	Pathogenic	[60]
23/20	c.7523G > A	p.Arg2508His	No	E 47	BSol	Pathogenic	[20, 55]
24/21	c.9293G > T	p.Ser3098Ile	No	E 63	BSol	Pathogenic	This paper
	c.14645C > T	p.Thr4882Met		E 101	Pore	Pathogenic	[76]
25/22	c.10097G > A	p.Arg3366His	AD	E 67	BSol	Likely pathogenic	[67]
	c.11798A > G	p.Tyr3933Cys		E 86	CSol	Pathogenic	[67, 69]
26/23	c.11708G > A	p.Arg3903Gln	No	E 85	CSol	Pathogenic	[55]
27/24	c.11708G > A	p.Arg3903Gln	AR	E 85	CSol	Pathogenic	[55]
	c.11708G > A	p.Arg3903Gln		E 85	CSol	Pathogenic	[55]
28/24	c.11708G > A	p.Arg3903Gln	AR	E 85	CSol	Pathogenic	[55]
	c.11708G > A	p.Arg3903Gln		E 85	CSol	Pathogenic	[55]
29/25	c.13724A > C	p.Asn4575Thr	No	E 94	pVSD	Pathogenic	[77]
30/26	c.13910C > T	p.Thr4637Ile	No	E 95	pVSD	Pathogenic	[78]
31/27	c.14209C > T	p.Arg4737Trp	AD	E 98	pVSD	Pathogenic	[79, 80]
32/27	c.14209C > T	p.Arg4737Trp	AD	E 98	pVSD	Pathogenic	[79, 80]
33/28	c.14582G > A	p.Arg4861His	No	E 101	Pore	Pathogenic	[66, 78]
34/29	c.14680G > C	p.Ala4894Pro	AD	E 102	Pore	Pathogenic	[81]
35/29	c.14680G > C	p.Ala4894Pro	AD	E 102	Pore	Pathogenic	[81]
36/29	c.14680G > C	p.Ala4894Pro	AD	E 102	Pore	Pathogenic	[81]
37/29	c.14680G > C	p.Ala4894Pro	AD	E 102	Pore	Pathogenic	[81]
38/30	c.14690G > A	p.Gly4897Asp	No	E 102	Pore	Pathogenic	[82, 83]
39/31	c.14693 T > C	p.Ile4898Thr	AD	E 102	Pore	Pathogenic	[83–85]
40/31	c.14693 T > C	p.Ile4898Thr	AD	E 102	Pore	Pathogenic	[83–85]
41/32	c.14693 T > C	p.Ile4898Thr	AD	E 102	Pore	Pathogenic	[83–85]
42/33	c.14693 T > C	p.Ile4898Thr	No	E 102	Pore	Pathogenic	[83–85]
43/34	c.14695G > A	p.Gly4899Arg	AD	E 102	Pore	Pathogenic	[85, 86]
44/35	c.14818G > A	p.Ala4940Thr	AD	E 103	Pore	Pathogenic	[78]
45/36	c.14928C > G	p.Phe4976Leu	AR	E 104	CTD	Pathogenic	[87]
	c.14928C > G	p.Phe4976Leu		E 104	CTD	Pathogenic	[87]
46/36	c.14928C > G	p.Phe4976Leu	AR	E 104	CTD	Pathogenic	[87]
	c.14928C > G	p.Phe4976Leu		E 104	CTD	Pathogenic	[87]
47/37	c.472_474delGAA	p.Glu158del	No	E 6	NTD-A	Pathogenic	This paper
48/38	c.1690 T > C	p.Tyr564His	No	E 16	NTD-C	Benign	[85]
	c.2930C > T	p.Thr977Met		E 24	RY1&2	Pathogenic	dbSNP: rs375865052
49/39	c.3866G > A	p.Arg1289Gln	No	E 28	SPRY2/SPRY3	Pathogenic	This paper
50/40	c.3901C > T	p.Arg1301Cys	No	E 28	SPRY2/SPRY3	Pathogenic	dbSNP: rs745920741
	c.5360C > T	p.Pro1787Leu		E 34	JSol	Benign	[56]
51/41	c.3935C > T	p.Pro1312Leu	No	E 28	SPRY2/SPRY3	Benign	This paper
52/42	c.4949 T > C	p.Leu1650Pro	No	E 34	SPRY2/SPRY3	Pathogenic	This paper
	c.6352C > T	p.Arg2118Trp		E 39	JSol	Pathogenic	[88]
	c.14918C > T	p.Pro4973Leu		E 104	CTD	Likely pathogenic	[79]
53/43	c.6178G > T	p.Gly2060Cys	No	E 38	JSol	Benign	[54, 56]
	c.13691G > A	p.Arg4564Gln		E 94	pVSD	Pathogenic	[7]
54/44	c.6352C > T	p.Arg2118Trp	AR	E 39	JSol	Pathogenic	[88]
	c.14918C > T c.10347 + 1G > A	p.Pro4973Leu IVS68 + 1G > A		E 104 I 68	CTD –	Likely pathogenic Pathogenic	[79] [88]
55/45	c.6377G > A	p.Arg2126Gln	No	E 39	JSol	Pathogenic	[89]
	c.11813G > A	p.Gly3938Asp		E 86	CSol	Pathogenic	[89]
	c.15022G > C	p.Glu5008Gln		E 106	CTD	Pathogenic	This paper
56/46	c.6617C > T	p.Thr2206Met	No	E 40	BSol	Pathogenic	[60]
	c.10537A > G	p.Thr3513Ala		E 71	BSol	Pathogenic	This paper
57/47	c.9145C > T	p.Leu3049Phe	No	E 61	BSol	Pathogenic	This paper
58/48	c.10516C > A	p.Gln3506Lys	No	E 71	BSol	Pathogenic	This paper
59/49	c.11609-2A > G	IVS83-2A > G	AD	I 83	–	Uncertain significance	This paper
60/49	c.11609-2A > G	IVS83-2A > G	AD	I 83	–	Uncertain significance	This paper
61/50	c.13952A > G	p.His4651Arg	AD	E 95	pVSD	Pathogenic	dbSNP: rs118192139
62/50	c.13952A > G	p.His4651Arg	AD	E 95	pVSD	Pathogenic	dbSNP: rs118192139
63/51	c.14815G > T	p.Asp4939Tyr	AD	E 103	Pore	Pathogenic	This paper
64/51	c.14815G > T	p.Asp4939Tyr	AD	E 103	Pore	Pathogenic	This paper
65/52	c.12063insAC c.2709_2711delCCC	p.Asp4021GlufsX4 p.His903del	AR	E 88 E 22	CSol RY1&2	Pathogenic Pathogenic	This paper This paper
66/53	c.7080insG	p.Pro2361AlafsX2	No	E 44	BSol	Likely pathogenic	This paper
67/54	c.14510delA	p.Gln4837ArgfsX3	AD	E 100	Pore	Pathogenic	[56]
68/54	c.14510delA	p.Gln4837ArgfsX3	AD	E 100	Pore	Pathogenic	[56]
69/55	c.4711A > G	p.Ile1571Val	No	E 33	SPRY2/SPRY3	Likely benign	[62, 67]
	c.9407delT*	p.Leu3136Argfs		E 63	BSol	Pathogenic	This paper

^a^Variant classification based on literature or bioinformatic analyses. ID: patient’s number; AR: autosomal recessive inheritance; AD: autosomal dominant inheritance; E: exon; I: Intron; NTD-A: N-terminal domain A; NTD-B: N-terminal domain B; NTD-C: N-terminal domain C; BSol: bridge solenoid; JSol: junctional solenoid; SPRY1-SPRY3: SP1a/ryanodine receptor domain; *RY1&2*: RyR repeats pairs; CSol: core solenoid; pVSD: pseudo voltage sensor domain; CTD: C-terminal domain

### *RYR1* mutations

Sixty-eight different nucleotide variations in *RYR1* sequence were identified in 69 patients from 55 unrelated families. As expected, mutations were localized with higher frequency in the 3 hotspot regions, than in the rest of the sequence (p < 0.0001). Variations included 59 missense mutations, 3 splice site variants, 4 small frameshift insertion or deletion, and two in-frame deletion of a single amino acid (Table 1). Among the 68 mutations, 16 were novel (Table 1). Twenty-three patients carried more than one *RYR1* variant (Table 1); of these patients, 13 (57%) were isolated case, 2 (9%) patients had an autosomal dominant (AD) and 8 (35%) an autosomal recessive (AR) inheritance pattern. Unfortunately, parents of patients were not available for analyses and the phase of the mutations was not assessed, with the exception of patients #17 and #69 who carry mutations known to be transmitted *in cis* [77, 90]*.*

### Muscle histopathology

**Fig. 1:**
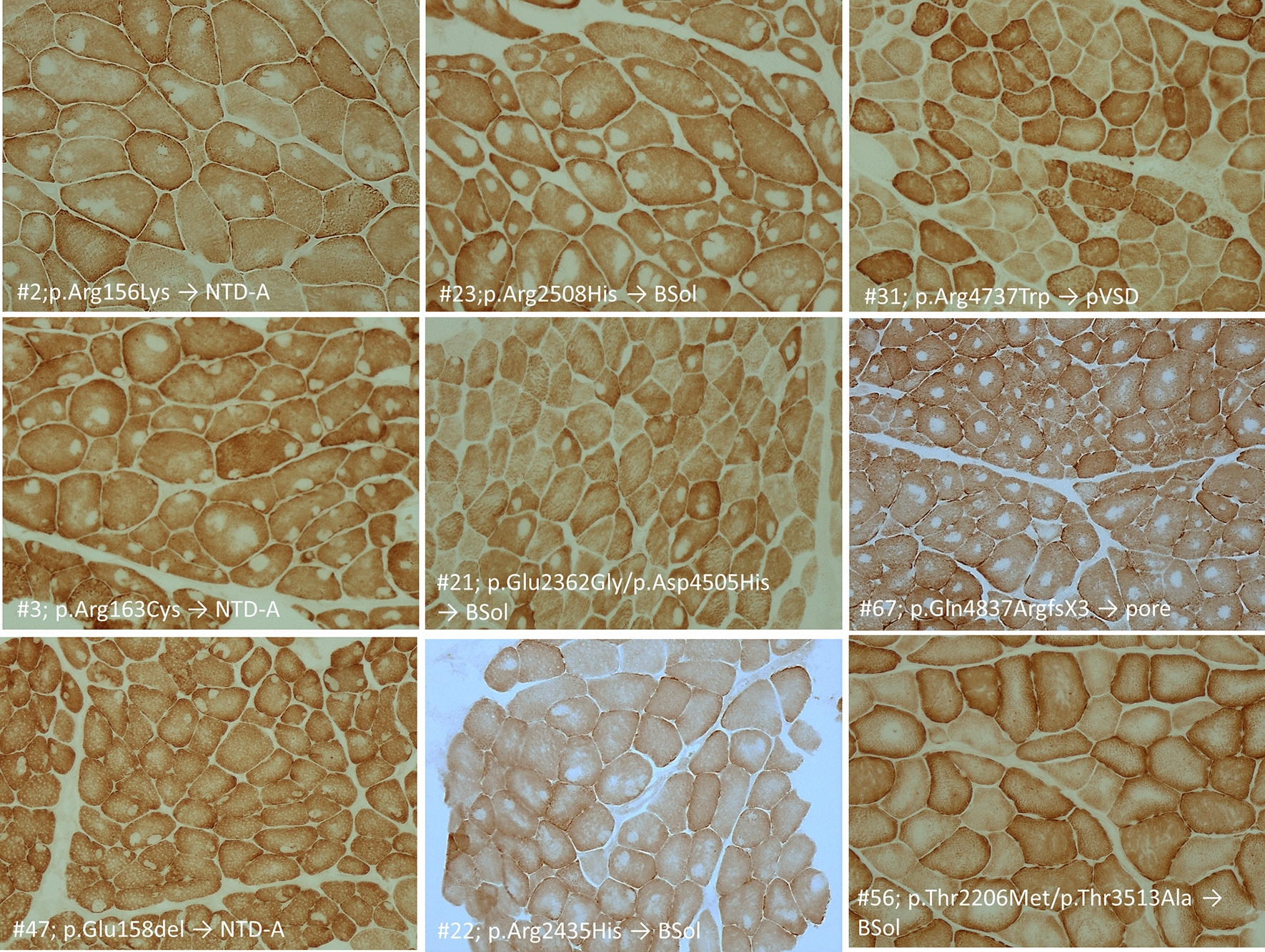
Muscle biopsy stained with COX from patients with core myopathy. In each panel is reported the patient #, the *RYR1* mutation and RyR1 protein domain where the mutation is located. Central core (CC) (single or multiple, centrally or peripherally located) are shown in patient #2, 3, 47, 23, 21, 22, 67 and 56. In patient #56 a minority of muscle fibers showed CC where minicores (Mm) (multiple, small randomly distributed areas with focal loss of mitochondrial activity) were more abundant. Patient #22 showed both CC and Mm where patient #31 muscle biopsy showed only Mm. Neither the *RYR1* mutation nor the RyR1 protein domain predict the ratio between CC and Mm

Muscle biopsy, done at time of diagnosis, was reviewed in 52/69 patients. Of the remaining 17 patients, 10 were familial cases where only a relative had the muscle biopsy done and in 7 patients muscle biopsy, consistent with core myopathy according to the referring physician, was not available for review. Quadriceps was biopsied in 45 patients, deltoid in 3, biceps and triceps in one each, while there was no information about the site of biopsy for 2 subjects. For all the samples, only transverse sections were available and analysed. All biopsies but one (#31, Additional file 3: T2) showed cores in a variable number of type 1 muscle fibres (ranging from 8 to 100%). Patient #31 had cores in 39% of type 2 muscle fibres only. In type 1 fibres, cores were centrally located (central cores, CC) in 67% of muscle biopsies, multiple minicores (Mm) and CC were present in 15% and Mm alone in 17% of muscle biopsies. CC and Mm were mostly observed in type 1 muscle fibres, whereas a minority of patients showed CC and Mm in type 2 fibres. No dusty cores were observed in this cohort of patients (Fig. 1).

Type 1 fibre predominance (defined as more than 45% of type 1 fibres) [91], ranging between 50–100%, was observed in 44 muscle biopsies (44/50; 88%) and increase in central nuclei (> 3% of fibres) was detected in 33 (33/50; 66%). An increase in perimysial and endomysial connective tissue was mild in 24 (24/51; 47%), severe in 6 (6/51; 12%) and absent in 20 (20/51; 39%) muscle biopsies. Only one biopsy (#44, Additional file 3: T2) showed rods in hypotrophic fibres associated with CC. Two muscle biopsies were obtained from patient #14. The first, performed at 5 years of age, showed no cores while the following biopsy, done 7 years later, showed CC in 100% of type 1 fibres (data not shown).

### Clinical description

**Fig. 2:**
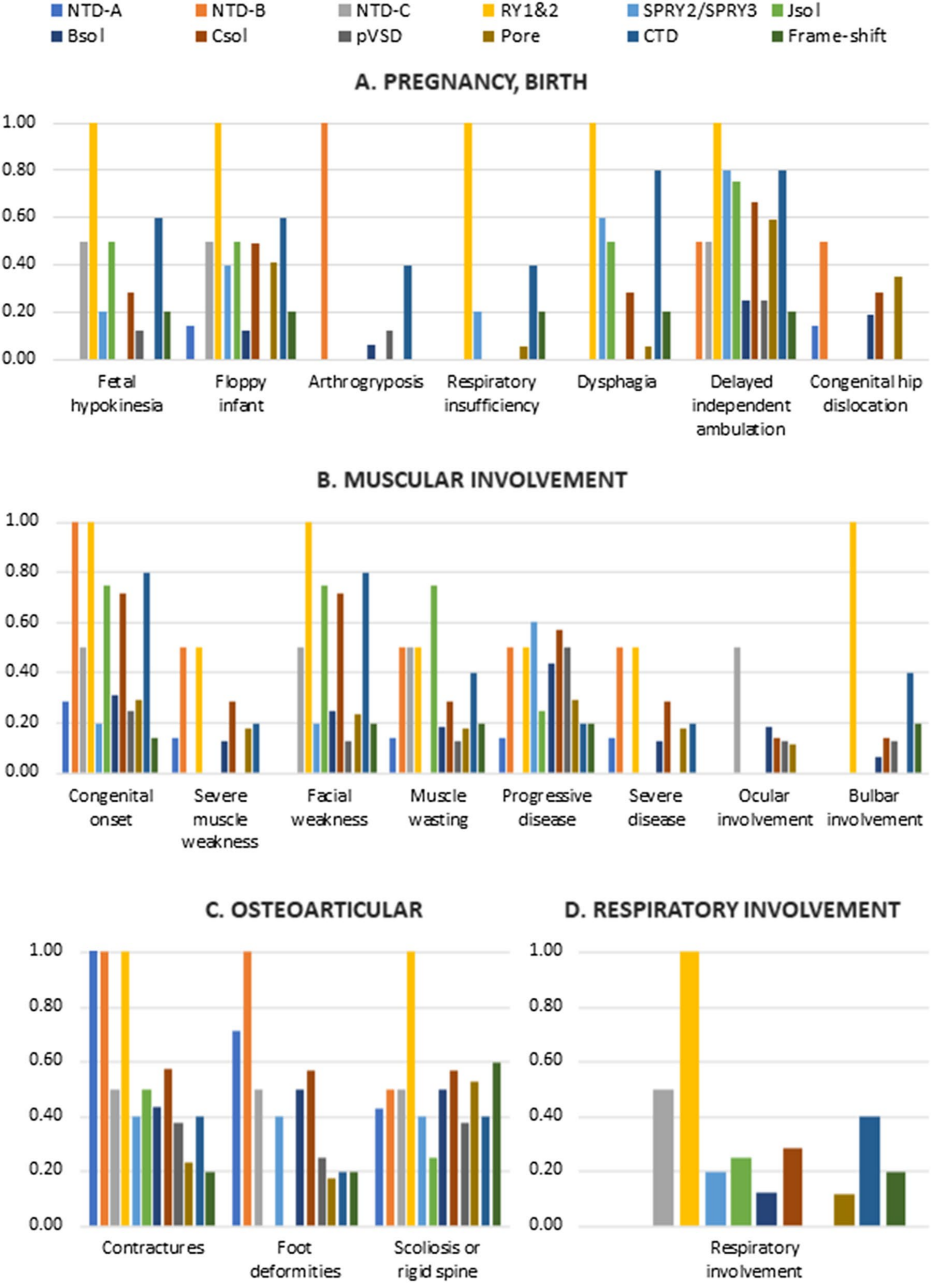
Histogram showing the proportion of patients manifesting specific phenotypes. Patients are grouped based on the mutated domain; each domain is colored following the legend on the top of the figure. A. Manifestations during pregnancy or at the birth. B. Muscular manifestations. C. Osteoarticular manifestations. D. Respiratory involvement

The studied cohort comprised 27 (39%) males and 42 females, aged from 2 to 72 years at the time of the last neurological evaluation (Additional file 4: T3). Thirty-eight/69 (55%) patients had a positive family history: 8 (8/38; 21%) AR and 30 (30/38; 79%) AD; family history was not available for 31 individuals (Table 1). In order to verify if mutations located in specific RyR1 domains were associated with more severe phenotype, we grouped patients based on the domain affected by the *RYR1* mutation and utilized clinical data at last evaluation for comparison. Subjects carrying more than one pathogenic mutation were described in each mutated domain group (Fig. 2 and Additional file 2: T1). Variations predicted or known to be benign were excluded from the analysis. Frame-shift mutations have been studied as separate group, independently from the affected domain. Results are summarized in Fig. 2 and Additional file 2: T1, and detailed clinical data are reported in Additional file 3: T2.

- NTD-A domain mutations. In our cohort 7 patients had mutations in NTD-A domain, only one of them (patient #47) had a severe clinical course. She presented at birth as a floppy infant, independent ambulation was acquired at 16 months and at 12 years of age the patient had a severe proximal and distal muscle weakness. Patient #1 had a congenital onset with hip dislocation, but an overall mild clinical course and at age 16 years the patient has a mild myopathy and retains the ability to walk and run. Muscle weakness was reported by 57% of patients but disease progression was stationary in all but one. The association between mutations in NTD-A with facial weakness and delayed independent ambulation was nominally significant (Fisher exact test p = 0.039, p = 0.038, respectively).
- NTD-B domain mutations. Two patients had mutations in NTD-B domain. Both patients presented at birth with arthrogryposis. Patient #7 had a severe AR myopathy with proximal and distal muscle weakness but was last seen at 3 years of age, while patient #8, who had delayed independent ambulation at 24 months of age had a subsequent slowly progressive disease and at age 57 years she is still ambulant with a moderate myopathy. The association between the presence of NTD-B mutation and arthrogryposis was nominally significant (Fisher exact test p = 0.013).
- NTD-C domain mutations. Two patients had mutations in this domain. These patients had an early or congenital onset disease, with steady progression and severity ranging from mild to moderate. Muscle wasting, facial weakness, ophthalmoparesis, scoliosis and respiratory involvement were present in 1/2 patients.
- Ry1&2 domain mutations. Two patients (#48, #65) carried a pathogenic *RYR1* mutation in the Ry1&2 domain. Both patients presented as floppy infant with dysphagia at birth. The patients had a mild to severe disease with bulbar involvement and respiratory insufficiency. Mutations in this domain were associated with foetal hypokinesia (p = 0.031), respiratory insufficiency (p = 0.007) and bulbar involvement (p = 0.010).
- SPRY2/SPRY3 domain mutations. Five sporadic patients belong to this group. Early onset and slowly progressive or steady disease characterize this subset of patients. One patient was diagnosed because of CK elevation and at 48 years-of-age remains paucisymptomatic (patient #50).
- JSol domain mutations. Four patients carried mutations in JSol domain. Two of them presented with foetal hypokinesia and delayed independent ambulation, 2/6 presented as floppy infant with dysphagia. All showed muscle weakness, 3/4 facial weakness, and 3/4 muscle wasting.
- BSol domain mutations. Sixteen patients had mutations in BSol domain. The onset was congenital in 5, early in 6, adult in 3 and in 2/16 asymptomatic patients hyperCKemia prompted diagnosis. Two patients had a severe weakness and one (patient #57) required not-mechanical ventilation. Ocular involvement was shown by 3/16 patients. Presence of mutations in this domain was associated with delayed independent ambulation (p = 0.008) and foetal hypokinesia (p = 0.030).
- CSol domain mutations. Seven patients had mutations in the CSol domain. The onset was congenital in 5 and early in 2patients. The severity of the disease was variable ranging from mild to severe. Six/7 patients had proximal muscle weakness and 5 also facial muscles involvement. Two patients had a severe, slowly progressive, myopathy. Contractures, foot deformities and scoliosis were frequent.
- pVSD domain mutations. Eight patients belonged to this group. Two patients had an adult onset and 6/8 had a congenital or early onset disease. A positive association with hypotonia at birth (“floppy infant” presentation, p = 0.043) was observed. Five patients had a moderate myopathy, 2 a mild myopathy, and only one patient was paucisymptomatic. Only one patient (#53) had a neonatal onset and at age 2 is still not ambulant.
- Pore domain mutations. Seventeen patients carried mutations in the pore domain. Seven presented as floppy infant, and 6 with congenital hip dislocation. Thirteen patients had a congenital (6) or early onset (7), one patient was identified through family screening and was asymptomatic at 1 < 6 years of age and for 2 patients no information were available regarding disease onset. Eight had a mild, 5 moderate and 3 a severe myopathy. Four patients showed facial muscle weakness, and 3 muscle wasting. Nine patients had scoliosis. There was a statistical association with foetal hypokinesia (p = 0.027), presence of contractures (p = 0.023), and foot deformities (0.010).
- CTD domain mutations. Five patients carried CTD domain mutations. The onset was congenital in 4 and early in one patient. The myopathy was moderate in 4 patients and severe in 1. Four patients showed facial weakness and 2 muscle wasting. Mutations in this domain were associated with foetal hypokinesia (p = 0.038) and dysphagia (p = 0.006).
- Frame-shift *RYR1* mutations. We identified 5 patients, belonging to 4 families, carrying frame-shift mutations. The onset of symptoms was heterogeneous comprising congenital (1), early (2), and adult (2). Muscle weakness was moderate in 2, mild in 4 and one patient was paucisymptomatic. Two patients had a moderate myopathy, and one of them had also rhinolalia and dysphagia.

### *RYR1* mutations bioinformatic analyses

Four RyR1 chains assemble to form a single calcium release channel and multiple 3D structures derived from cryomicroscopy studies describing their organization are available in the literature. To investigate the effect of mutations in our dataset, we generated a homology model of the human RyR1 monomer using the orthologous 3D structure from rabbit (identities = 96.91%, coverage = 72%). Resulting model presents an overall good geometry with 90.09% of residues occupying the Ramachandran favoured regions. The 2.05% of residues are estimated to occupy unfavourite positions. Visual inspection shown that most of them localize at the borders of regions predicted as intrinsically disordered. Pairing these data with a number of bad bonds corresponding to 0.01%, we suggest that our model is good enough to assess the effect of mutations found in patients. In details, mutations were classified as “pathogenetic from literature” if already reported so, “pathogenetic from function” based on in silico studies, “likely benign” based on literature data and/or bioinformatics analyses, and “truncating, frameshift and intronic” based on their effect on the protein sequence.

#### *RYR1* mutations “pathogenetic from literature”

Thirty-eight mutations were identified in this subset.

**Fig. 3:**
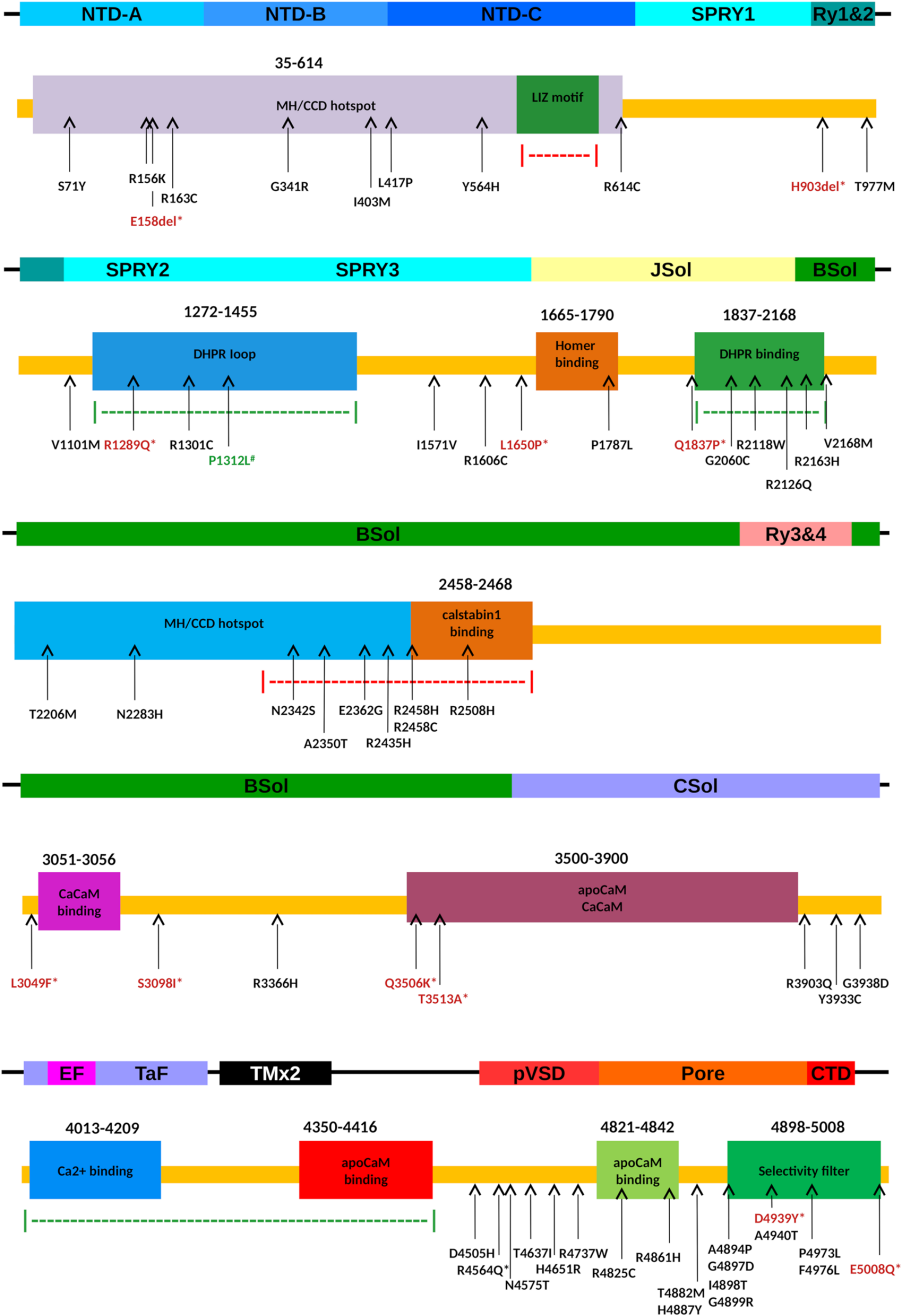
Schematic representation of RyR1 monomer functional domains. RyR1 sequence is presented as yellow bar with functional regions represented as colored boxes. Domain structural organization is presented on top. Dotted lines highlight regulative regions where red implies inhibition and green activation of the channel. Arrows represent the position for each mutation (frameshift mutations not shown). New mutations modelled in silico are wrote in red and marked with * when predicted as pathogenic, in green marked with # when benign

**Fig. 4:**
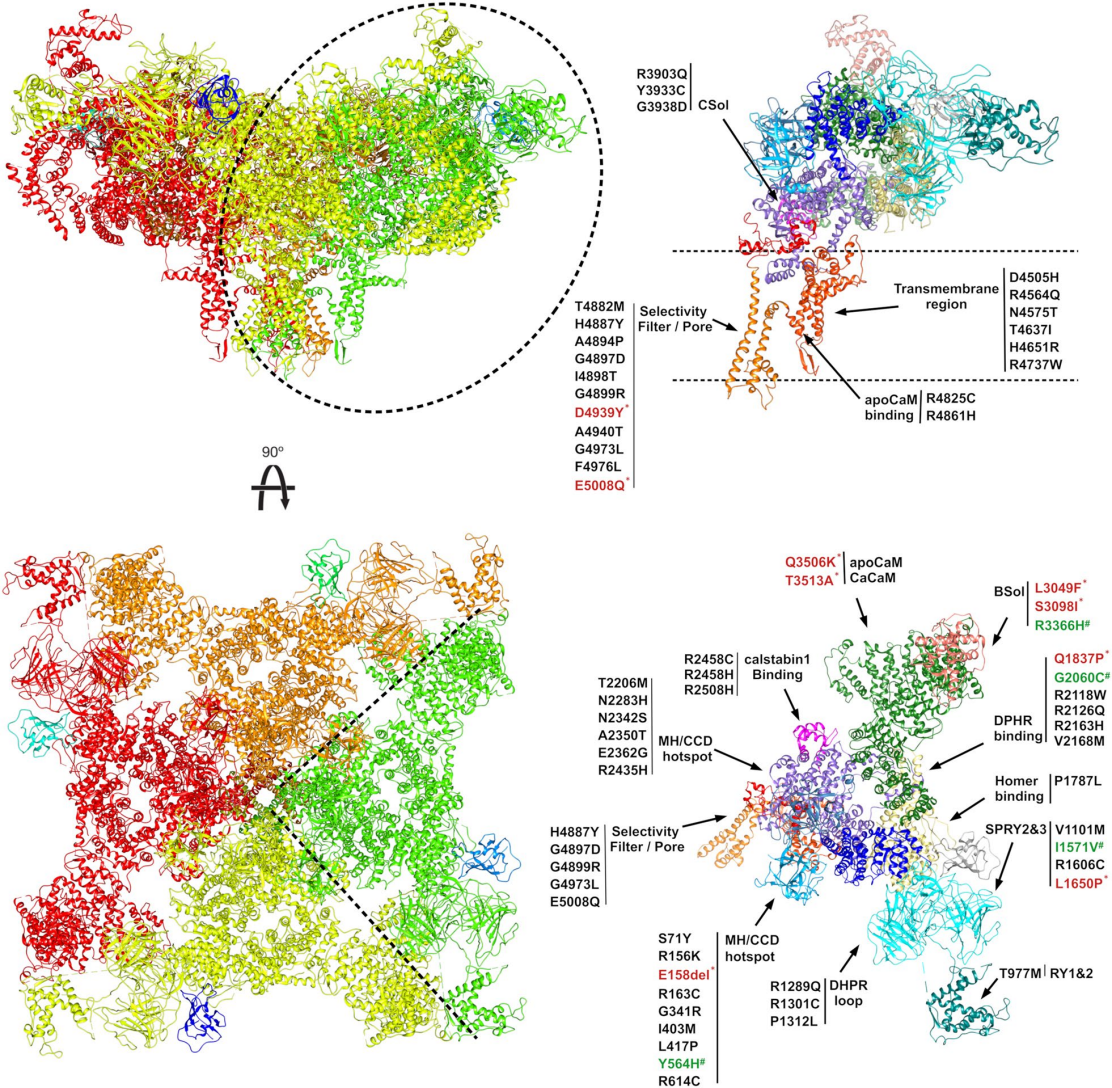
Cartoon representation of the human RyR1 structure. Front and top views of RyR1 tetramer assembly. Different colors represent each monomer. On right side, isolated RyR1 monomer colored by functional domains with mutations noted and grouped accordingly (frameshift mutations not shown). New mutations modelled in silico are wrote in red and marked with * when predicted as pathogenic, in green marked with # when benign

- NTD-A, NTD-B, and NTD-C domains. Mutations falling into RyR1 N-terminal region (amino acid 35–614) are causative of both MH and CCD [92]. We found 7 known mutations in this region (p.Ser71Tyr [54, 55], p.Arg156Lys [56], p.Arg163Cys [57, 58], p.Gly341Arg [59, 60], p.Ile403Met [61], p.Leu417Pro [62], p.Arg614Cys [63, 64]). Structural investigations of these variants suggested that they may promote destabilization of MIR folding domain, thus predisposing to pathological phenotypes (Figs. 3, 4). According to literature, the region between amino acids 1272–1455 is responsible for interaction with the II-III loop of DHPR, which is an intrinsically unstructured protein acting as calcium channel gating activator [93]. Previous experimental validation demonstrates that this region is essential for skeletal muscle contraction in vivo [94]. We hypothesize that the mutations in this region promote secondary structure rearrangement impairing the DHPR function.
- JSol, BSol and CSol domains. The impairment of DHPR was was predicted also for p.Arg2118Trp [88] and p.Arg2126Trp [89], as these mutations occurs in a RyR1 region known to interact with DHPR as well [93]. The pathogenicity of p.Arg2118Trp may also be due to the mutation’s proximity to the calmodulin binding site on RyR1 [88]. Mutations p.Arg2163His [57], p.Val2168Met [60], p.Thr2206Met [60] and p.Asn2283His [54] are located in a conserved region of RyR1 central domain. Functional studies demonstrated that these mutations result in increased caffeine sensitivity and altered calcium handling in cells [54, 57, 60]. RyR1 has other regulative modules such as the 2350–2458 segment, known to promote the channel inactivation [95, 96]. The effect of mutations in this region is related to the loss of interaction with calstabin1, a cis–trans peptidyl-prolyl-isomerase, required for the physiological gating of the channel [95]. Due to its specific function, mutations occurring in this region, such as p.Asn2342Ser [71], p.Ala2350Thr [73], p.Glu2362Gly [55], p.Arg2435His [60], p.Arg2458Cys [68], p.Arg2458His [68] and p.Arg2508His [20, 55] are predicted to be likely pathogenetic, due to their possible ability to inactivate the channel gating. Moreover, stability predictors evaluated all the variants as destabilizing. Several predictors found p.Arg3366His [67] and p.Tyr3933Cys [67] to be pathogenic, both mutations involving conserved RyR1 regions. In particular, p.Tyr3933Cys resides in the RyR/IPR3 homology associated domain which is involved in Ca2 + regulation [67]. A similar effect may be hypothesized also for p.Arg3903Gln [55].
- pVSD, Pore and CTD domains. The C-terminal region of RyR1 is known to bind CaM and to act as selectivity filter, regulating the number and chemical property of passing ions [97, 98]. The region forms a transmembrane domain and amino acids mutations in this area resulted commonly as pathogenic prone, disrupting the channel activity [98]. As expected [16, 98], six transmembrane helices were correctly predicted between residues 4285–4302, 4329–4346, 4351–4368, 4563–4580, 4794–4811 and 4838–4855. The region between residues 4300–4850 is known to be an important regulative element for RyR1 activation/inactivation switch [98–100], with several known pathological mutations falling within. The analysis of six mutations (i.e. p.Asp4505His [74, 75], p.Asn4575Thr [77], p.Thr4637Ile [78], p.His4651Arg [78], p.Arg4737Trp [79, 80] and p.Arg4825Cys [66]) in the interval 4505–4825 (Figs. 2, 3), suggested a potentially pathological phenotype, affecting the apoCAM binding domain functionality and the ions flux balance regulation. A detailed study of the effect of p.Asp4505His is reported by Groom et al. [75]. This mutation affects the RYR1-divergent region 1 (D1; amino acids 4254–4631) [101], deletion of the majority of this region (Δ4274–4535) potentiates voltage-gated Ca2 + release and enhances channel’s sensitivity to activation by DHPR [102]. Thus, the p.Asp4505His mutation may enhance RyR1 release-channel sensitivity to activation by disrupting the integrity of the D1-negative regulatory module [75]. We found several mutations in RyR1 transmembrane region that had been previously associated to core myopathies, i.e. p.Arg4861His [66, 78], p.Thr4882Met [76], p.His4887Tyr [72], p.Ala4894Pro [81], p.Gly4897Asp [82, 83], p.Ile4898Thr [83–85], p.Gly4899Arg [85, 86], p.Ala4940Thr [78], p.Pro4973Leu [79] and p.Phe4976Leu [87].

#### 3.2.2 “Pathogenetic from function” *RYR1* mutations

We modelled fifteen variants, which were either novel, or not previously studied (11 variants) or with unclear effect on the RyR1 channel from literature (4 variants) (i.e. p.Val1101Met [65], p.Gly3938Asp [89], p.Arg4564Gln [7], p.Arg1606Cys [70]). The human RyR1 (Uniprot code: P21817) was analysed with a collection of different bioinformatics tools to evaluate the impact of amino acid mutations on the RyR1 protein function.

- NTD-A domain. The mutation p.Glu158del localizes in a flexible loop connecting two β-strands at the beginning of the MIR2 domain. Pathogenicity predictors suggest that this mutation reduces the domain stability. Further, multiple mutations in this area are already known to promote both MH and CCD [92].
- RY1&2 domain. The mutation p.Thr977Met was predicted as damaging. Thr977 is exposed to the solvent and structural investigation suggests its variation in methionine to destabilize the correct RY1&2 (amino acids 850–1054) domain folding. Previous observations proposed that this repeated region, together with the SPRY domains, acts as calstabin1 interacting region [103]. A pathological phenotype 1n patients harbouring the p.Thr977Met may be associated with impairment of this interaction.
- SPRY2/SPRY3 domain. The p.Val1101Met is predicted as probably damaging as Val1101 engages hydrophobic interactions with Phe1089 and Phe1091 stabilizing the SPRY2 domain spanning residues 1055–1241. According to literature, the region within residues 1272–1455 is responsible for interaction with the II-III loop of DHPR [94]. Experimental validation demonstrated that the region is relevant for skeletal muscle contraction in vivo [94], suggesting that variants p.Arg1289Gln and p.Arg1301Cys may interfere with the DHPR-mediated regulation of RyR1. A damaging effect on the protein structure is predicted for p.Arg1606Cys. Structural inspection shows Arg1606 to form electrostatic interactions with both Asp1111 and Glu1113. At the structural level, our analysis suggests that this mutation alter correct localization of the SPRY2 domain inducing local protein unfolding. Damaging effect is also predicted for p.Leu1650Pro. This position is well conserved among eukaryotes indicating it may play a relevant structural and/or functional role. Our structural investigation showed Leu1650 to form multiple van der Waals interactions with residues from the adjacent chain, suggesting that its mutation can negatively affect RyR1 tetramerization, yielding a functionally reduced channel. Mutation p.Gln1837Pro localizes in a short alpha helix segment connecting two longer helices spanning residues 1803–1852. The entire segment is part of a larger junctional solenoid domain. As proline is known to destabilize secondary structure, we believe the mutation induces a local unfolding of this domain.
- BSol and CSol domains. Mutations p.Leu3049Phe, p.Lys3098Ile, p.Gln3506Lys and p.Thr3513Ala localize in a long disordered region important for RyR1 association with CaM (Fig. 2). All of them are predicted as damaging, possibly interfering with CaM mediated RyR1 regulation. A different pathogenic effect is predicted for p.Gly3938Asp. The Gly3938 localizes at the internal binding interface between RyR1 monomers directly facing the residue Glu79 from an adjacent monomer chain. The repulsive interaction introduced by p.Gly3938Asp is rather predicted to lower RyR1 complex stability than having a negative effect on monomer structure.
- pVSD, Pore and CTD domains. The region between residues 4300–4850 is known to be an important regulative element for the RyR1 activation/inactivation switch [98–100], with several known pathological mutations falling within. In particular, p.Arg4564Gln impairs an electrostatic interaction between Arg4564 and Tyr4792. The analysis of mutations localizing into the interval 4505–5008, i.e. p.Asp4939Tyr and p.Glu5008Gln, predict them as damaging and suggest a potentially pathological phenotype, affecting the apoCAM binding domain functionality and the ions flux balance regulation. The evidence is also in agreement with [104].

#### 3.2.4 “Likely benign” *RYR1* mutations

- NTD-A domain. In silico pathogenicity prediction suggests p.Tyr564His to be tolerated. A modest structural impact is also predicted by investigating the residue interacting network around Tyr564 (Fig. 3). Indeed, investigation shows p.Tyr564His to localize in the N-terminal solenoid spanning residues 393–627. Based on our model, this residue is partially exposed and modestly involved in the tandem repeat domain folding. We thus suggest this mutation to be likely neutral.
- SPRY2/SPRY3 domain. As for p.Tyr564His, sequence conservation analysis shows that Pro1312 is only limitedly conserved among different species, reinforcing the idea that mutation p.Pro1312Leu may be tolerated. Another possible explanation of this result is that stability predictors fail to address p.Pro1312Leu pathogenicity as Pro1312 localizes in an intrinsically disordered region. The p.Ile1571Val [62, 67] variant is reported to be benign.
- JSol domain and intronic region. p.Pro1787Leu [56], p.Gly2060Cys [54, 56, 105], and c.10259 + 7G > A are reported to be benign variants.

#### Truncating and frameshift *RYR1* mutations

We found four frameshift mutations i.e. p.Pro2361AlafsX2, p.Leu3136Argfs, p.Gln4837ArgfsX3 and p.Asp4021GlufsX4. In our series, a predicted altered quantitative level of RyR1 is consistent with stable or slowly progressive mild/moderate myopathy, suggesting that the level of RyR1 protein may be critical for the normal excitation–contraction coupling [56, 60]. The c.10347 + 1G > A mutation had already been described as probably damaging. The mutation likely abolishes the donor splice site, producing a shorter or unstable mRNA and it was predictably damaging by in silico analyses [88]. Finally, we found also a new intronic variant i.e. c.11609-2A > G, localized in a highly conserved region. Different bioinformatic tools predicted that the mutation causes the loss of a splicing-acceptor site in intron 83. The mutation likely leads to the production of an aberrant transcript with the partial out-of-frame retention of intron 84.

## Discussion

In this paper we collected clinical, histological and molecular data from a large cohort of core myopathies patients carrying *RYR1* mutations with the intent to use in silico modelling to evaluate the possible pathogenetic impact of the identified *RYR1* variants, and to verify if variants targeting to specific RyR1 domain are associated to a more severe phenotype.

We used a candidate gene approach using *RYR1* Sanger sequencing to screen a large cohort of core myopathy patients. One hundred fifty-three patients were enrolled and *RYR1* mutations were identified in 69 (45%) of them. The lack of detailed histopathological information in most of the original cohort of patients, did not allow to assess the overall prevalence of *RYR1* mutations in core and multi-minicore diseases. Nevertheless, since CCD is reported to be mainly due to *RYR1* mutation [20], while MmD are associated with mutations in variety of genes (*RYR1*, but also *SEPN1*, and less frequently in *MYH2, UNC45B, MYH7*, *TTN*, *MEGF10*, *SECISBP2*, *ACTA1*, *ACTN2*, *CCD78*, and *FXR1*) [8] we can hypothesize in our original cohort a majority of MmD patients carrying mutations in genes other than *RYR1*. The mutational approach in our study has some limitations. First, we can not rule out deep intronic or non-canonical splice site variants, since the primers were designed to cover only exons and canonical splice sites. With our methods the non-canonical splicing events, that are emerging to be numerous and often tissue specific, have been missed and therefore also their potential role in core myopathies may be under recognized. Second, we cannot exclude to have missed some *RYR1* variants because of some intrinsically pitfalls in Sanger sequencing (i.e. the quality of sequence in the first 15 to 40 bases where the primers bind, or quality of sequencing in long reads, etc.), and finally, *RYR1* Sanger sequencing does not allow to look for mutations in other known genes possibly involved in core myopathies. The knowledge of all deleterious alleles in a given patients may contribute to the understanding of the role of the mutational load in disease phenotype expression.

The cohort of patients studied confirmed clinical heterogeneity in *RYR1*-related core myopathy, even if the selection criteria we used to enrol patients in this study were chosen to obtain a homogeneous group of patients adding to the presence of *RYR1* mutations a histopathological diagnosis of core myopathy.

*RYR1*-related AD CCD presents with a variable clinical spectrum including congenital (47%), early (38%) and adult (14%) onset manifestations. A delay in reaching motor milestones was frequently described (30 patients), and only one patient is still not walking at the last evaluation at 2 years of age. In patients with adult onset, symptoms and signs at clinical presentation included cramps and myalgias, fatigue and muscle weakness prevalent in the lower limbs.

Distribution of weakness was symmetrical, both proximal and distal, in the pelvic and shoulders girdles in 19% of patients and in a minority included also axial muscle weakness. Facial weakness, configuring the typical myopathic face in congenital myopathy, was present in 35% of patients. Spine and joint contractions were frequent in our cohort.

As previously reported [56, 62, 106], patients with recessive *RYR1* mutations (8) were overall more severe than AD cases. Onset was congenital in all, arthrogryposis (37.5% vs 0%), floppy infant presentation (62.5% vs 17%), respiratory insufficiency (37.5% vs 0%) and hip dislocation (29% vs 15%) at onset were more frequent in AR than AD cases (Additional file 3: T2). However, even in the AR cases disease progression was variable (stable in 4 patients, slowly progressive in 3 and one patient showed improvement over time). At last evaluation (range 3–40 years) all patients were ambulant but one, who was able to walk only with support at 16 years of age. Overall disease phenotype was severe/moderate with frequently associated foot deformities (50%) and scoliosis (75% of patients).

Thirty-one patients in our cohort were isolated cases. Unfortunately, parents were not available to unequivocally establish the inheritance pattern. Based on the inheritance pattern of previously reported mutations 3 cases were likely AR CCD (i.e. patient #1 carrying two mutations previously reported i*n trans* [54]; patient #9 [62] and 38 [82] carrying a *RYR1* mutation previously reported in AR cases in compound heterozygosity [62, 82]), and 6 likely AD CCD (i.e. patient #8 [61], #14 carrying a mutation previously reported in severe AD centronuclear myopathy [70], #29 [77], #30 and #33 harbouring mutations previously reported in core/rod myopathy [75] and in congenital myopathy with uniformity of type 1 muscle fibres [80] and patient #42 [83]). Three patients carried *RYR1* mutations previously associated to AD MH (i.e. #2 [55], #16 [57] and #26 [55]) (Table 1; Additional file 3: T2). In the remaining cases either novel mutations or previously unreported association of various *RYR1* mutation do not allow to infer the phase of the variants. All 3 putative AR cases had a congenital onset and mild to severe myopathy associated with contractures or scoliosis according to the rule of more severe phenotype in AR vs AD cases.

It is interesting to note that at least 15 patients in our cohort carried mutations associated to MH. MH is usually linked to minimal or absent muscle weakness and muscle biopsy may, or may not, show cores. Given our selection criteria, all patients presented cores at muscle biopsy and 3/15 showed moderate and 6/15 mild muscle weakness. Two patients (#16 and #26) presented a congenital onset (with arthrogryposis, floppiness, hip dislocations in patient #16 and foetal hypokinesia, dysphagia in patient #26), delayed independent ambulation (30 and 24 months respectively) and an overall mild myopathy with stationary progression over time but associated with joint contractures, scoliosis and pes cavus. In these two patients, given the severe clinical picture, it is likely that a second *RYR1* mutation was not detected. Only two patients were asymptomatic (in one diagnosis was reached because of a positive family history and in the second in the work up of a hyperCKemia) and two were paucisymptomatic (myalgia, cramps). Among patients carrying mutations previously associated to MH, at least three had a MH reaction during anaesthesia: patient #3 during pes cavus surgery, patient #21 during surgery for congenital muscular torticollis and patient # 23 during spinal arthrodesis for scoliosis. Patient #3 and #21 had an early onset moderate myopathy with typical features of congenital myopathy, where patient #23 was paucisymptomatic and was studied for hyperCKemia. Given the selection bias in our cohort of patients we were not able to assess either the percentage of MH patients presenting with muscle weakness nor the percentage showing core at histopathology. It is possible to speculate that the presence of core at muscle biopsy predisposes to the development of muscle weakness, but a large number of patients is needed to reach a definite conclusion. On the other hand, patients in our cohort may simply express the clinical variability associated to *RYR1* mutations. For example, family 3, carrying the well-known p.Arg163Cys mutation associated with MH, displayed a wide phenotypic spectrum. Among the members of this family, one individual (patient #3) presented with an early onset moderate myopathy with muscle wasting, contractures, foot deformities and scoliosis, patient#4 had an adult onset mild myopathy, while patient #5 came to medical attention for asymptomatic hyperCKemia.

The reasons of this intrafamilial variability are not known but genetic modifiers in genes other than *RYR1* may be responsible for the clinical variability.

Five patients (belonging to four families) in our cohort carried heterozygous small frame-shift deletion or insertion mutations causing a premature stop codon predicted to result in a reduced level of RyR1. Recently, it has been shown in a knocked-in mouse for a *RYR1* single allele frame-shift mutation, that the level of *RYR1* transcript and RyR1 protein are decreased without affecting the other SR proteins. The mouse also showed a mild reduction of muscle performance and decreased muscle strength, suggesting that hypomorphic *RYR1* mutation should result in a mild clinical phenotype because of a functional reserve of RyR1 protein [107]. Indeed, all six patients followed the postulated rule of a mild reduction in muscle strength.

The *RYR1* gene carries a large number of polymorphisms as well as causative mutations: there are 1,783 public variants vs 843 unique public variants reported in LOVD database (https://databases.lovd.nl/shared/genes/RYR1; accessed June 2021) making the assessment of pathogenicity of the identified variant a crucial issue. We identified 68 *RYR1* variants and 16 were novel.

We used an integrative in silico approach to evaluate the pathogenic impact of some of the *RYR1* mutations identified. Our approach included an in-depth literature search along with a systematic bioinformatics analysis of some of the *RYR1* variants identified that allowed us to determine the molecular details supporting the pathogenicity of several novel *RYR1* mutations. Despite our promising data, several hurdles have to be considered: the lack of the human RyR1 crystal structure paired with the presence of large intrinsically disordered regions, the large size of the protein and its composition of multiple functional and regulative elements, all pointing to a difficult assessment of a single variant.

In heterozygous patients the protein may be constitute of variable proportion of mutated monomers, the ratio wild-type/mutated monomers could be directly linked to the disease severity but assessing stoichiometry in single patient it is not feasible at the moment. On the other hand, different mutations lead to different pathogenic mechanisms, such as haploinsufficiency, inactivation of regulative sites, impairment of complex assembly and alterations in the channel’s gating properties. To add to the complexity, the genetic background may modify the pathological effect of the *RYR1* mutations. For example, variations in other genes associated with core myopathies or known to be RyR1 interactors (i.e. *FKBP1B*, *TRDN*, *ASPH*, *FKBP1A*, *STAC3*, *CACNA1S*, *CACNA1C*, *CACNA1A*, *NOS1*, *CALM1*) or even other unknow genes may present single nucleotide polymorphism that cause subtle modification on the protein product but sufficient to modify the phenotype. This hypothesize phenomenon is well represented by the phenotypical variability among member of the same family. Finally, as mentioned before, epigenetic modifications of *RYR1* may also play a role [108] or the methylation status of genes implicated in cytosolic Ca^2+^ buffering or trafficking of the Na^+^/Ca^2+^ exchanger [109].

The difficult interpretation of the pathogenicity of *RYR1* variant is reported also by Johnston and colleagues, who focused their attention on *RYR1* mutations causing malignant hyperthermia. They revised the American College of Medical Genetics and Genomics and the Association for Molecular Pathology (ACMP/AMP) criteria for variant interpretation highlighting the difficulty to define general rules to estimate the effect of a mutations [110].

From a myopathological point of view the majority of patients (67%) showed at muscle biopsies core lesions in type 1 muscle fibres, 17% of patients had multiple minicores in both type 1 and type 2 muscle fibres and 15% of muscle biopsies showed both central core and multiple minicores. In a subset of patients, the presence of cores and minicores was confirmed with ultrastructural electron microscopy (EM) studies but EM was not formally investigated in all patients. No correlations were detected among the number of fibres with core and clinical severity. It is interesting to note that the only patient who had 2 muscle biopsy done at age 5 and 12 years showed a dramatic modification of muscle morphology. The first muscle biopsy did not show cores while the biopsy taken at 12 years of age disclosed central core in 100% of type 1 fibres. These observations are in line with the hypothesis that core lesions may not represent a primary developmental abnormality but could be secondary to a maintained abnormal contraction caused by any excitation–contraction coupling defect as previously reported [23].

## Conclusions

Considering all the limitations detailed above we were not able to correlate specific genotype to phenotype, but focusing on protein domains we suggest that, in our cohort, mutations in some domains are more frequently associated with specific phenotypes. For example, the pore domain is associated with foetal hypokinesia, contractures and foot deformities. Moreover, it is of interest that all patients (#48 and #65) carrying mutations in Ry1&2 presented with particularly severe phenotype, as this domain is associated with foetal hypokinesia, bulbar involvement and respiratory insufficiency. However, the low number of patients do not allow definitive conclusions at this point.

## Supplementary Information

**Additional file 1**: Bioinformatics pipeline used for mutations effect prediction.

**Additional file 2**: Affected RyR1 domains and clinical description.

**Additional file 3**: Histological description of core myopathies patients.

**Additional file 4**: Patients *RYR1* mutations and clinical description.

## Data Availability

All data generated or analysed during this study are included in this published article [Table 1, Additional file 2: T1, Additional file 3: T2, Additional file 4: T3].

## Abbreviations

RyR1: Ryanodine receptor 1 protein
*RYR1*: Ryanodine receptor 1 gene
CCD: Central Core Disease
MmD: Multi-minicore Disease
MH: Malignant hyperthermia
DHPR: Dihydropyridine receptor
NTD-A: N-terminal domain A
NTD-B: N-terminal domain B
NTD-C: N-terminal domain C
BSol: Bridge solenoid
JSol: Junctional solenoid
CSol: Core solenoid
SPRY: SP1a/ryanodine receptor domain
RY1&2: RyR repeats pairs 1 and 2
RY3&4: RyR repeats pairs 3 and 4
TaF: Thumb and forefingers domain
TMx: Transmembrane helices
pVSD: Pseudo voltage sensor domain
CTD: C-terminal domain
*SEPN1*: Selenoprotein N1 gene
*SELENON*: Selenoprotein N1 gene
NADH-TR: Nicotinamide adenine dinucleotide dehydrogenase-tetrazolium reductase
COX: Cytochrome C oxidase
SDH: Succinate dehydrogenase
AD: Autosomal dominant
AR: Autosomal recessive
CC: Central cores
Mm: Multiple minicores
