# Supplementary figures and images for: Expanding the clinical-pathological and genetic spectrum of RYR1-related congenital myopathies with cores and minicores: an Italian population study
Source: Acta Neuropathol Commun. 2022 Apr 15;10:54. doi: 10.1186/s40478-022-01357-0 (PMC9013059; doi:10.1186/s40478-022-01357-0)

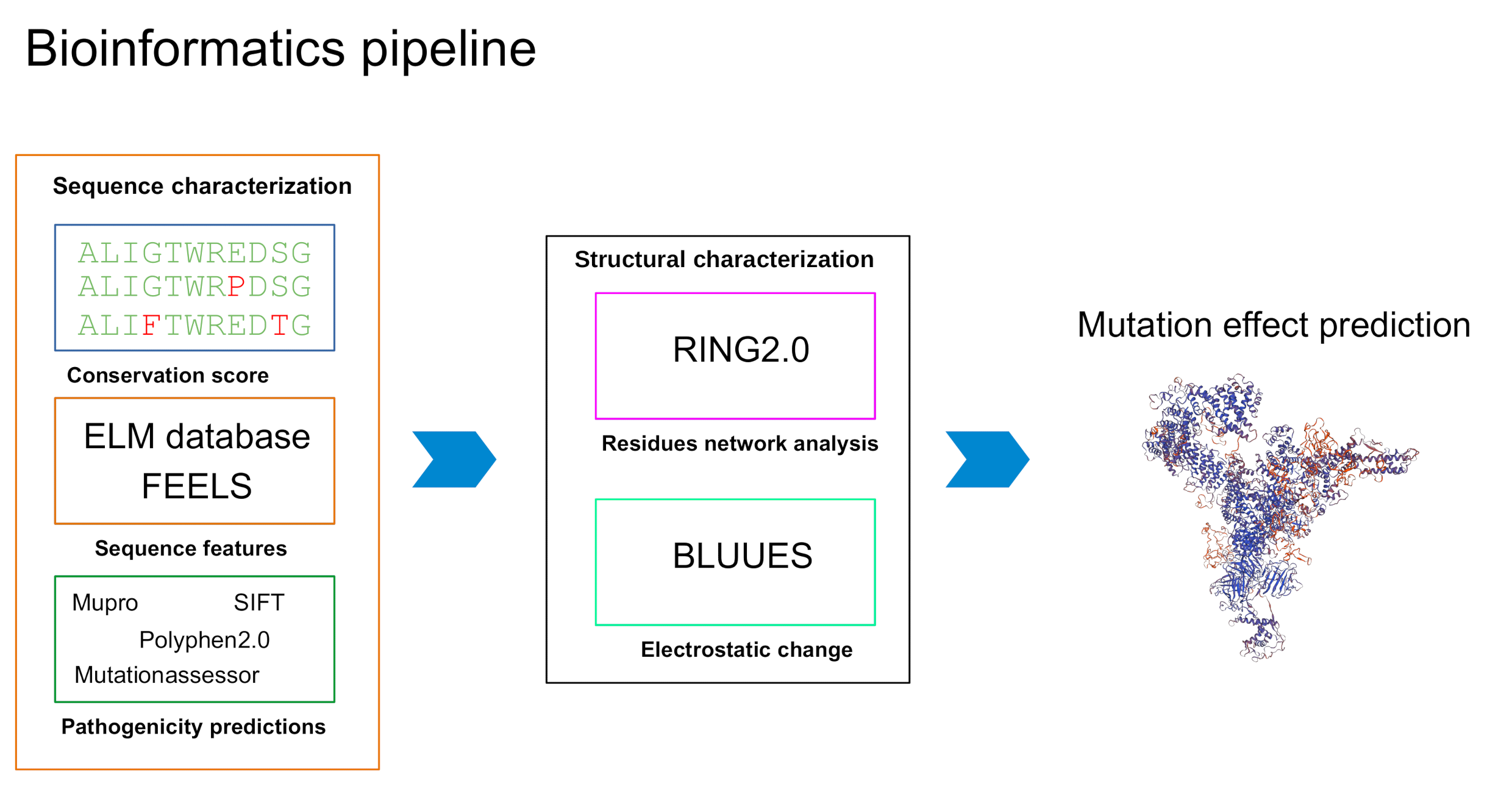

Supplement: Supplementary file 1 — Additional file 1: Bioinformatics pipeline used for mutations effect prediction. [file 40478_2022_1357_MOESM1_ESM.tiff]
